# Data on the experiments of temperature-sensitive hydrogels for pH-sensitive drug release and the characterizations of materials

**DOI:** 10.1016/j.dib.2018.01.042

**Published:** 2018-01-31

**Authors:** Wei Zhang, Xin Jin, Heng Li, Run-run Zhang, Cheng-wei Wu

**Affiliations:** State Key Laboratory of Structure Analysis for Industrial Equipment, Department of Engineering Mechanics, Dalian University of Technology, Dalian 116024, China

## Abstract

This article contains experimental data on the strain sweep, the calibration curve of drug (doxorubicin, DOX) and the characterizations of materials. Data included are related to the research article “Injectable and body temperature sensitive hydrogels based on chitosan and hyaluronic acid for pH sensitive drug release” (Zhang et al., 2017) [1]. The strain sweep experiments were performed on a rotational rheometer. The calibration curves were obtained by analyzing the absorbance of DOX solutions on a UV–vis-NIR spectrometer. Molecular weight (*M*_*w*_) of the hyaluronic acid (HA) and chitosan (CS) were determined by gel permeation chromatography (GPC). The deacetylation degree of CS was measured by acid base titration.

**Specifications Table**

**Table:** 

Subject area	Chemistry
More specific subject area	Polysaccharide Chemistry
Type of data	Table, Figure
How data was acquired	Experiments were performed using rotational rheometer (Anton Paar MCR302, Austria), UV–vis-NIR spectrometer (Lambda 750s, PerkinElmer, USA),Malvern Viscotek GPC system (Viscotek TDA 305, USA) and GPC system (Shimadzu, Japan).
Data format	analyzed
Experimental factors	Solutions with varying HA content, DOX solutions of different pH
Experimental features	Strain sweep experiments, the calibration curves of drug, molecular weight of HA and CS, deacetylation degree of CS
Data source location	Dalian University of Technology, China
Data accessibility	Data are available with this article

**Value of the data**

● The strain sweep experiments ensure the accuracy for measurements of the rheological properties.
● The calibration curves provide information for the quantification of hydrogel-releasing drugs.
● The characterizations of polysaccharides provide basic standard data for researchers to study their protocols.

## 1 Data

This article contains experimental data on the strain sweep, the calibration curve of drug (doxorubicin, DOX) and the characterizations of materials of our paper [1].

**Fig. 1:**
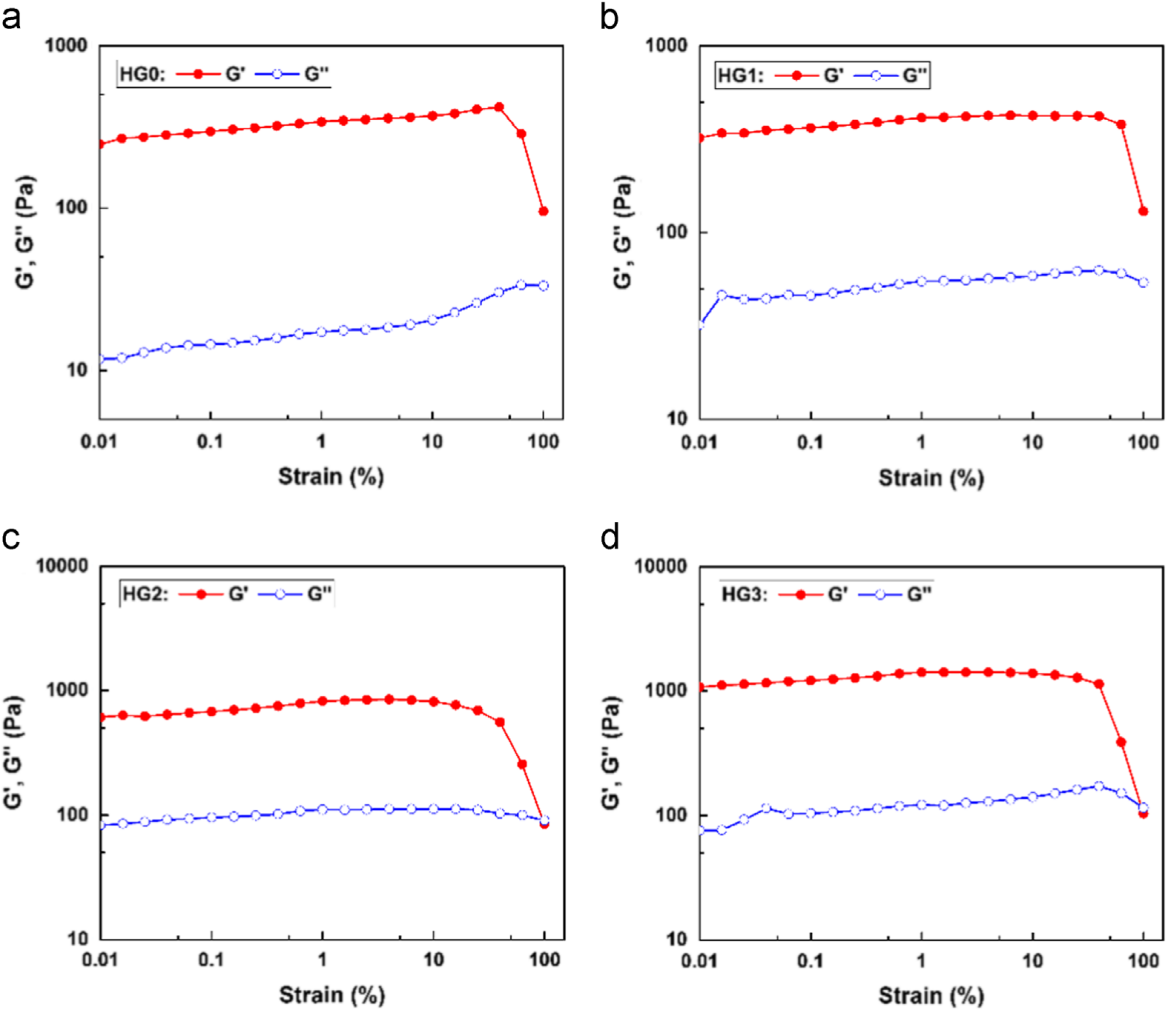
Change of *G*′ and *G*′′ with strain: (a) HG0, (b)HG1, (c) HG2, (d) HG3.

**Fig. 2:**
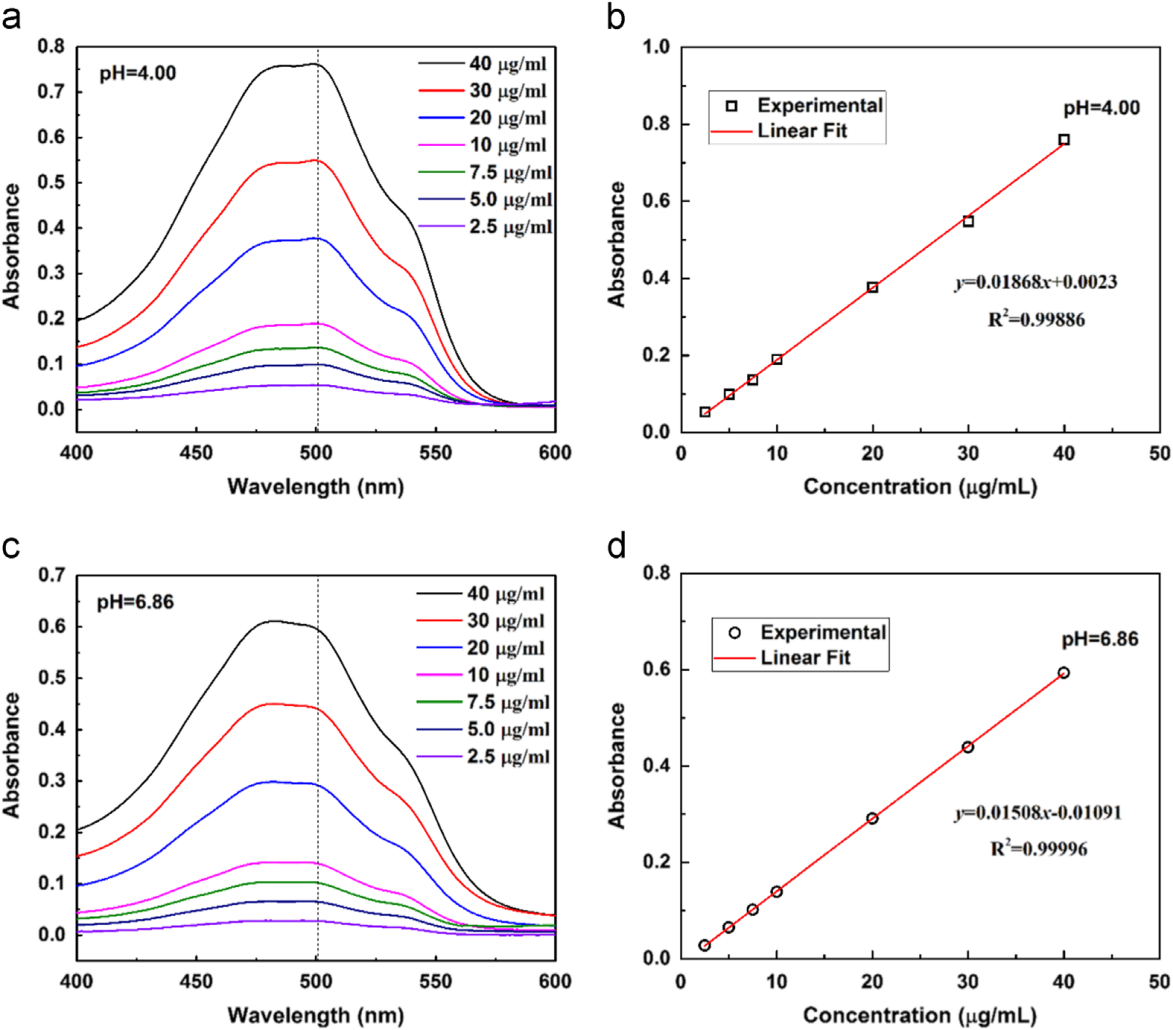
Ultraviolet absorption spectroscopy of DOX aqueous solutions (a,c) and the calibration curve of absorbance at 501 nm as a function of DOX concentration.

The data present the strain sweep of hydrogels with varying HA contents (Fig. 1) and the calibration curves of drug (Eqs. (1), (2) and Fig. 2).The molecular weight of the HA and CS was determined by GPC. The deacetylation degree of CS was measured by acid base titration (Table 1).

**Table 1:** Results of CS deacetylation degree.

Number of sample	NaOH volume (mL)	Water content (%)	Deacetylation degree (%)	Average value (%)	Standard deviation
1	7.70	10.3	80.5	81.3	0.865
2	7.02	10.0	82.2		
3	7.28	9.84	81.1		

## 2 Experimental design, materials and methods

### 2.1 Strain sweep experiments

The rheological properties were performed on a rotational rheometer (Anton Paar MCR302, Austria) fitted with a PP25 plate indenter - platform plate configuration. The CS/HA/GP were piped between the plates, and mineral oil was used to cover the marginal surface of the solution for preventing water evaporation during the tests. The strain sweep experiments were carried out and the results are plotted in Fig. 1. It can be found that for all the hydrogels investigated, both *G*′ and *G*′′ are independent of strain when the strain is less than 10%.

### 2.2 Calibration curve of drug (DOX)

DOX powder was dissolved in pH 4.00 and pH 6.86 phosphate-buffered saline solution to make DOX solution at various concentrations ranging from 2.5 μg/mL to 40 μg/mL. The absorption spectra were recorded using UV–vis-NIR spectrometer and the maximum absorbance (at 501 nm) for different concentrations of DOX was used to plot the calibration curve. The absorption spectra in pH 4.0 and pH 6.86 are given in Fig. 2a and c, respectively and the corresponding calibration curves are given in Fig. 2b and d.

At pH 4.00, the calibration curve can be expressed using Eq. (1).

(1) $$y=0.01868x+0.0023\left(R^{2}=0.99886\right)$$

At pH 6.86, the calibration curve can be expressed using Eq. (2).

(2) $$y=0.01508x-0.01091\left(R^{2}=0.99996\right)$$

### 2.3 Molecular weight of HA

Molecular weight (*M*_*w*_) of the HA was determined by gel permeation chromatography (GPC) [2] using a Malvern Viscotek GPC system equipped with a refractive index detector, light scattering and viscometer detectors (Viscotek TDA 305, USA). The HA powder was dissolved in deionized water to prepare 0.5 mg/mL solution. The sample was measured at a flow-rate of 0.8 mL/min and a column temperature of 35 °C. Molecular weight of the HA is 1.26 × 10^6^ and polydispersity index (d=Mw/Mn) is 1.34.

### 2.4 Molecular weight of CS

Molecular weight of the CS was determined by GPC [3]. The CS powder was dissolved in 2% acetic acid solution. The solution at a flow rate of 0.6 mL/min and TSK-gel GMPWXL column from TOSOH CORPORATION (Japan) equipped with RID 20 refractive index detector was used for this determination. Molecular weight of the CS is 8.75 × 10^4^ and polydispersity index (d=Mw/Mn) is 1.99.

### 2.5 Deacetylation degree of CS

The deacetylation degree of CS was measured by acid base titration [4]. 0.1 mol/L 30 mL hydrochloric acid (HCl) was added in 0.5 g CS sample, 2 drops of methyl orange indicator were added to the solution. 0.1 mol/L sodium hydroxide (NaOH) was titrated until the red color of the indicator changed into yellow. 0.5 g CS was dried at 105 °C for 12 h to measure moisture content. Degree of deacetylation was determined by the following equations:

(3) $$\mathrm{Amino}\;\mathrm{content}=\left(C_{1}V_{1}-C_{2}V_{2}\right)\times 0.016/G(100-W)\times 100\%$$

(4) Degreeofdeacetylation=Aminocontent(%)/9.94%×100%

where, $C_{1}$ (mol/L) is the concentration of HCl; $C_{2}$ (mol/L) is the concentration of NaOH; $V_{1}$, the volume of HCl (mL); $V_{2}$, the volume of NaOH (mL); G (g) is the weight of CS; W (%) is the water content of CS. The results are shown in Table 1.
