# Supplementary material for: Data on the experiments of temperature-sensitive hydrogels for pH-sensitive drug release and the characterizations of materials
Source: Data Brief. 2018 Jan 31;17:419–23. doi: 10.1016/j.dib.2018.01.042 (PMC5988482; doi:10.1016/j.dib.2018.01.042)
Supplement: Supplementary file 1 — Transparency document [file mmc1.zip › COI_DIB_2109_V1.docx]

COI - All author declares there is no Conflict of Interset
